# Manufacture of Clinical-Grade CD19-Specific T Cells Stably Expressing Chimeric Antigen Receptor Using *Sleeping Beauty* System and Artificial Antigen Presenting Cells

**DOI:** 10.1371/journal.pone.0064138

**Published:** 2013-05-31

**Authors:** Harjeet Singh, Matthew J. Figliola, Margaret J. Dawson, Simon Olivares, Ling Zhang, Ge Yang, Sourindra Maiti, Pallavi Manuri, Vladimir Senyukov, Bipulendu Jena, Partow Kebriaei, Richard E. Champlin, Helen Huls, Laurence J. N. Cooper

**Affiliations:** 1 Division of Pediatrics, Children's Cancer Hospital, The University of Texas MD Anderson Cancer Center, Houston, Texas, United States of America; 2 Department of Stem Cell Transplantation and Cellular Therapy, The University of Texas MD Anderson Cancer Center, Houston, Texas, United States of America; 3 The University of Texas Graduate School of Biomedical Sciences at Houston, Houston, Texas, United States of America; Wayne State University, United States of America

## Abstract

Adoptive transfer of T cells expressing a CD19-specific chimeric antigen receptor (CAR) is being evaluated in multiple clinical trials. Our current approach to adoptive immunotherapy is based on a second generation CAR (designated CD19RCD28) that signals through a CD28 and CD3-ζ endodomain. T cells are electroporated with DNA plasmids from the *Sleeping Beauty* (SB) transposon/transposase system to express this CAR. Stable integrants of genetically modified T cells can then be retrieved when co-cultured with designer artificial antigen presenting cells (aAPC) in the presence of interleukin (IL)-2 and 21. Here, we reveal how the platform technologies of SB-mediated transposition and CAR-dependent propagation on aAPC were adapted for human application. Indeed, we have initiated clinical trials in patients with high-risk B-lineage malignancies undergoing autologous and allogeneic hematopoietic stem-cell transplantation (HSCT). We describe the process to manufacture clinical grade CD19-specific T cells derived from healthy donors. Three validation runs were completed in compliance with current good manufacturing practice for Phase I/II trials demonstrating that by 28 days of co-culture on γ-irradiated aAPC ∼10^10^ T cells were produced of which >95% expressed CAR. These genetically modified and propagated T cells met all quality control testing and release criteria in support of infusion.

## Introduction

A chimeric antigen receptor (CAR) recognizes cell-surface tumor-associated antigen independent of human leukocyte antigen (HLA) and employs one or more signaling molecules to activate genetically modified T cells for killing, proliferation, and cytokine production [1]. Targeting CD19 has been achieved by us and others through the enforced expression of a CAR that recognizes CD19 independent of HLA. Gene therapy can be combined with immunotherapy to redirect the specificity of T cells for B-lineage antigens and patients with advanced B-cell malignancies benefit from infusion of such tumor-specific T cells [1]–[9]. In contrast to other groups that genetically modify T cells using recombinant retrovirus, we have developed a non-viral gene transfer approach to enforce expression of the introduced CAR. This was achieved using the *Sleeping Beauty* (SB) system which we adapted for human application [10], [11]. SB-mediated gene transfer consists of coordinated excision and insertion of SB transposon from a plasmid by the SB transposase into TA dinucleotide repeats in the target-cell genome [12], [13]. To improve therapeutic potential, our 2^nd^ generation CAR [14] signals through CD28 and CD3-ζ with the expectation that this will sustain T-cell proliferation and recycle effector functions *in vivo*. To retrieve T-cell integrants stably expressing the CAR we developed aAPC to select for T cells *in vitro* that are capable of sustained CAR-mediated propagation. These aAPC (designated clone #4) co-express CD19 along with the co-stimulatory molecules CD86, CD137L, a membrane-bound mutein of IL-15, and the Fc-receptor CD64.

The SB system and aAPC have been combined to generate CD19-specific CAR^+^ T cells in support of multiple clinical trials under INDs at MD Anderson Cancer Center (MDACC) [15]. To improve the graft-versus-tumor (GVT)-effect, we have used these two platform technologies to generate genetically modified T cells for infusion after autologous (IND# 14193) and allogeneic HSCT (IND# 14577), including after umbilical cord blood transplantation (IND# 14739), and lymphodepleting chemotherapy (IND# 15180). This report describes the manufacturing processes and associated testing to generate the clinical products for use in these investigator-initiated trials [16].

Our clinical-grade CD19-specific T cells, prepared in compliance with current good manufacturing practice (cGMP) for Phase I and II trials can be generated by (i) electrotransfer of supercoiled DNA plasmids derived from SB system coding for CAR as a transposon and (ii) numeric expansion on CD19^+^ aAPC clone #4. The manufacturing process includes every-7-to-10-day additions of γ-irradiated aAPC in the presence of soluble recombinant human IL-2 and IL-21. After 28 days, typically at least 90% of the propagated T cells express CAR and are cryopreserved for infusion. These T cells meet release criteria defined by sterility, phenotype, viability, and cell number. In-process testing reveals that the electroporated/propagated T cells express CAR in a memory/naïve population, have a normal karyotype, preserved TCR Vβ repertoire, and are able to lyse CD19^+^ tumor targets in a CAR-dependent manner.

## Materials and Methods

### Generation of clinical-grade DNA plasmids

The SB transposon, _CoOp_CD19RCD28/pSBSO, expresses the human codon optimized (CoOp) 2^nd^ generation _CoOp_CD19RCD28 CAR under EF-1/HTLV hybrid composite promoter (InvivoGen) comprised of Elongation Factor- 1α (EF-1α) [17] and 5′ untranslated region of the Human T-Cell Leukemia Virus (HTLV) [11], [18]. The derivation of this DNA plasmid is described in **Figure S1**. The SB transposase, SB11, under the cytomegalovirus (CMV) promoter is expressed in *cis* from the DNA plasmid pCMV-SB11 [11]. The derivation of this DNA plasmid is described in **Figure S2**. Both plasmids were sequenced in their entirety and manufactured by Waisman Clinical Biomanufacturing Facility (Madison, WI) using kanamycin for selection of the bacterial strain *E. Coli* DH5α. The release criteria for the DNA plasmids are shown in **Table S1**. CD19 was expressed using the DNA plasmid ΔCD19_CoOp_-F2A-Neo/pSBSO (**Figure S3**).

### Cell counting

Trypan-blue exclusion was used to distinguish live from dead cells and counted using Cellometer (Nexecelom Bioscience) [11].

### Isolation of PBMC

Leukapheresis products from two male volunteer healthy donors were purchased from Key Biologics LLC (Memphis, TN). The peripheral blood mononuclear cells (PBMC) were isolated by our adapting the Biosafe Sepax system (Eysins, Switzerland) for work in compliance with cGMP. Briefly, after closing all the clamps on the CS-900 kit, 100 mL Ficoll (GE Healthcare) was aseptically transferred via 60 mL syringes to a density gradient media bag (“ficoll bag”) via Luer-lock connector and the tubing was heat sealed using a hand held sealer (Sebra, Model# 2380). The kit was spike-connected to a 1,000 mL bag containing CliniMACS buffer (PBS/EDTA, Miltenyi, Cat#70026) with 20 mL 25% Human Serum Albumin (HSA) (Baxter) (2% v/v, wash buffer) for washes, a final product bag [300 mL Transfer Pack with Coupler (Baxter/Fenwal 4R2014)] and a reagent/blood bag. Using the density gradient-based separation protocol (v126), the syringe piston was loaded into the centrifuge chamber and the cover of the Sepax unit closed. The reagent/blood bag along with product bags was hung and all stopcocks were seated on the rotary pins in the ‘T’ position. After connecting the pressure-sensor line, the kit was validated by automatic single-use test and then manually primed using gravity flow. After completion of the cycle, the final product was aseptically transferred into a centrifuge tube and washed once each with wash buffer and phosphate buffered saline (PBS) at 400 g for 10 minutes. After counting, cells were cryopreserved using cryopreservation media (50% HSA, 40% Plasmalyte, 10% DMSO) in CryoMACS Freeze bags (Miltenyi) and vials (Nunc) using BM5 program (4°C to −4°C at rate −2°C/min, −4°C to −60°C at rate −35°C/min, −60°C to −20°C at rate 8°C/min, −20°C to −45°C at rate −2.5°C/min, −45°C to −80°C at rate −10°C/min) in a controlled-rate freezer (Planer Kryo 750).

### Manufacture of aAPC (clone #4) master and working cell banks

K562 were transduced by lentivirus at the University of Pennsylvania to generate aAPC (clone #4, designated CJK64.86.41BBL.GFP.IL-15.CD19) that co-express (i) CD19, (ii) CD64, (iii) CD86, (iv) CD137L, and (v) membrane bound IL-15 (mIL-15) as a bi-cistronic vector with EGFP. The aAPC were numerically expanded in HyQ RPMI 1640 (Hyclone) containing 10% heat-inactivated defined FBS (Hyclone) and 2 mmol/L Glutamax-1 (Life Technologies-Invitrogen) culture media (CM) maintaining the cells at 5×10^5^/mL. A master cell bank (MCB) of 320 vials was produced through Production Assistance of Cellular Therapies (PACT) (**Table S2**). A 200 vial working cell bank (WCB) of Clone 4 aAPC derived from the MCB was then generated at MDACC and tested (**Table S3**).

### aAPC (clone #4) to selectively propagate CAR^+^ T cells

The γ-irradiated aAPC were used to numerically expand the genetically modified T cells. Thawed aAPC from WCB were propagated in CM for up to 60 days in VueLife cell culture bags and harvested using Biosafe Sepax II harvest procedure. Briefly, CS-490.1 kit was connected to a 300 mL output bag (transfer pack) via Luer lock connection. The separation chamber was installed in the pit and the tubing was inserted into the optical sensor and stopcocks aligned in T-position. After connecting the pressure sensor line, the product bag and supernatant/plasma bags were hung on the holder. The modified protocol PBSCv302 was selected from the Sepax menu and the volume of input product to be processed (initial volume) was set to ≤840 mL. After validation and kit test, the procedure was started. Following completion, the bags were removed, clamps closed and the kit was removed. The cells from the final product bag were aseptically removed, washed twice with wash media (10% HSA in Plasmalyte) and counted. aAPC were irradiated (100 Gy) using a CIS BIO International radiator (IBL-437 C#09433) and cryopreserved for later use in cryopreservation media using controlled-rate freezer (Planer Kryo 750).

### OKT3-loading of aAPC

The OKT3-loaded (via CD64) aAPC (clone#4) were used to propagate control (CAR^neg^) autologous control T cells that had not undergone genetic modification. The aAPC, obtained from culture, were incubated overnight in serum-free X-Vivo 15 (cat # 04-744Q, Lonza) containing 0.2% acetyl cysteine (Acetadote, Cumberland Pharmaceuticals) termed Loading Medium (LM). The next day cells were washed, irradiated (100 Gy) using a Gamma Cell 1000 Elite Cs-137 radiator (MDS Nordion), resuspended in LM at a concentration of 10^6^ cells/mL along with 1 µg/10^6^ cells of functional grade purified anti-human CD3 (clone-OKT3, 16-0037-85, eBioscience) and incubated with gentle agitation on a 3-D rotator (Lab-Line) at 4°C for 30 minutes. Following three washes with LM the cells were used in experiments or frozen in aliquots in liquid nitrogen in vapor layer for later use.

### Manufacture of CAR^+^ T cells

Thawed PBMC were resuspended in (i) Human T-cell kit (cat# VPA-1002, Lonza; 100 µL for 2×10^7^ cells in one cuvette), with (ii) the DNA plasmid (_CoOp_CD19RCD28/pSBSO) coding for CD19RCD28 CAR transposon (15 µg supercoiled DNA per 2×10^7^ PBMC per cuvette), and (iii) the DNA plasmid (pCMV-SB11) coding for SB11 transposase (5 µg supercoiled DNA per 2×10^7^ PBMC per cuvette). This mixture was immediately transferred to a cuvette (Lonza), electroporated (defining culture day 0) using Nucleofector II (Program U-14, Amaxa/Lonza), rested in 10% RPMI complete media for 2 to 3 hours, and after a half-media change, incubated overnight at 37°C, 5% CO_2_. The following day, cells were harvested, counted, phenotyped by flow cytometry, and co-cultured with γ-irradiated aAPC at a ratio of 1∶2 (CAR^+^ T cell:aAPC), which marked culture day 1 and the beginning of a 7-day stimulation cycle. IL-21 (cat # AF-200-21, PeproTech) and IL-2 (cat # NDC 65483-116-07, Novartis) were added on a Monday-Wednesday-Friday schedule onwards of day 1 and day 7 respectively. NK cells can prevent the numeric expansion of CAR^+^ T cells, especially if their overgrowth occurs early in the tissue culturing process. Therefore, a CD56-depletion was performed if CD3^neg^CD56^+^ cells ≥10% using CD56 beads (cat #70206, Miltenyi Biotech, 20 µL beads/10^7^ cells) on LS columns (cat #130-042-401, Miltenyi Biotech) in CliniMACS buffer containing 25% HSA (80 µL/10^7^ cells). T cells were cryopreserved as backup on culture day 21 after electroporation and the end of the 3^rd^ stimulation cycle using a controlled-rate freezer (Planer Kryo 750) as described above, and stored in liquid nitrogen (vapor-layer). The cell counts for total, CD3^+^, and CAR^+^ T cells were plotted over time and slopes determined using linear regression. The fold-expansion results were compared using Student's t-test. CD4/CD8 ratios were calculated for each time point and validation runs and averaged.

### Generation of CAR^neg^ control T cells

As a control, 5×10^6^ mock transfected PBMC were co-cultured with irradiated and anti-CD3 (OKT3) loaded K562-derived aAPC clone #4 at a ratio of 1∶1 in a 7-day stimulation cycle. All the cultures were supplemented with IL-21 (30 ng/mL) from culture day 1 onwards, and IL-2 (50 U/mL) starting 7 days after the start of the culture. All cytokines were subsequently added on a Monday-Wednesday-Friday schedule.

### Cell lines

CD19^+^ Daudi β_2_m [Burkitt lymphoma, co-expressing β_2_ microglobulin, [19]] and CD19^+^ NALM-6 (pre-B cell) were cultured as described previously [11]. EL-4 cells (mouse T-cell lymphoma line) from ATCC were modified to express CD19 using the construct ΔCD19_CoOp_-F2A-Neo/pSBSO. Briefly, 5×10^6^ EL-4 cells were resuspended in 100 µL of Amaxa Mouse T cell Nucleofector kit (Catalogue # VPA-1006) with SB transposon (ΔCD19_CoOp_-F2A-Neo/pSBSO, 3 µg) and SB transposase (pCMV-SB11, 1 µg) and electroporated (program X-001) using Nucleofector II (Lonza). The transfectants were cultured in a cytocidal concentration of G418 (0.8 mg/mL) and underwent fluorescent activated cell sorting (FACS) for homogeneous expression of CD19 to obtain a clone (clone #17). Jurkat cell were obtained from ATCC and electroporated (Program T-14, Nucleofector II, Lonza) with _CoOp_CD19RCD28/pSBSO using the Amaxa/Lonza Nucleofector solution (Kit V). Two weeks after electroporation, Jurkat cells stably expressing CAR underwent FACS for homogeneous expression of CAR to obtain a clone (clone #12) [20]. Cell lines were maintained in HyQ RPMI 1640 (Hyclone) supplemented with 2 mmol/L Glutamax-1 (Invitrogen) and 10% heat-inactivated Fetal Calf Serum (FCS) (Hyclone; 10% RPMI). All cell lines were validated using STR profiling or karyotyping according to institutional cell line authentication policy.

### Immunophenotype of cells

Cells were stained using antibodies (**Table S4**) in 100 µL FACS Buffer (2% FBS, 0.1% Sodium Azide) for 30 minutes at 4°C. For intracellular staining, after fixing/permeabilization for 20 minutes, cells were stained in perm wash buffer with appropriate antibodies for 30 minutes at 4°C. Acquisition was performed using FACSCalibur (BD Bioscience) and analyzed using Cell Quest (BD Bioscience) or FCS Express 3.00.0612 (De Novo Software, Thornhill, Ontario, Canada).

### Western Blot

Protein expression of the chimeric CD3-ζ (73-kDa) derived from CD19RCD28 was assessed as described previously [10]. Briefly, protein lysates were transferred using iBlot Dry Blotting System (Invitrogen) onto nitrocellulose membrane, incubated with mouse anti–human CD3-ζ monoclonal antibody (cat # 551033, 0.5 µg/mL, BD Biosciences, CA) followed by horseradish peroxidase (HRP)-conjugated goat anti-mouse IgG (cat # 1858413, 1∶10,000; Pierce, IL), developed using SuperSignal West Femto Maximum Sensitivity substrate (Pierce, IL) and chemiluminescence captured using VersaDocTM 4000 gel documentation system (BioRad, CA).

### Telomere Length Analysis by Fluorescence *In Situ* Hybridization and Flow Cytometry (Flow-FISH)

Telomere length of the T cells was measured by using the Telomere PNA Kit/FITC for Flow Cytometry (DAKO) according to the manufacturer's instructions. Briefly, isolated cells (CD4 or CD8) and control cells (cat # 85112105, CEM-1301 cell line; ECACC) were mixed in equal measure in hybridization solution with or without FITC-labeled telomere PNA probe for 10 minutes at 82°C; hybridized overnight in the dark at room temperature; washed twice with a wash solution at 40°C; resuspended in PBS containing 2% FCS and propidium iodide (1 µg/mL); and analyzed on a FACSCalibur (BD Biosciences). FITC-labeled fluorescent calibration beads (cat # 824A, Quantum™ FITC MESF, Bangs Laboratories) were used for calibration of the flow cytometry machine. Relative telomere length (RTL) was determined by comparing T cells with a CEM-1301 cell line per:
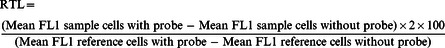



### Chromium Release Assay

T cells were evaluated for their cytotoxicity in a standard 4-hour chromium release assay using ^51^Cr-labeled target cells. T cells were plated in triplicate at 1×10^5^, 0.5×10^5^, 0.25×10^5^, 0.125×10^5^ and 0.0625×10^5^ cells/well with 5×10^3^ target cells in a 96-well V-bottom plate (Costar). After incubation, 50 µL of supernatant was harvested onto LumaPlate (Perkin Elmer), read in TopCount NXT (Perkin Elmer) and percent specific lysis was calculated per:
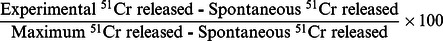
Spontaneous and maximum release was determined by measuring chromium in the conditioned supernatant from target cells incubated with CM or 0.1% Triton X-100 (Sigma), respectively.

### Endotoxin Testing

Endotoxin level in final products was determined using Endosafe® -PTS Portable Test System (Charles River Laboratories) as per the manufacturer's guidelines. The test has a detection limit of 0.01–10 EU/mL which can be converted to EU/patient weight.

### Mycoplasma testing

Mycoplasma detection by PCR was performed using the TaKaRa Mycoplasma Detection Kit (Clontech) according to manufacturer's instructions.

### T-cell receptor Vβ repertoire

T-cell receptor (TCR)-Vβ usage of culture day 28 and day 35 CAR^+^ T cells was determined using a panel of 24 TCR Vβ–specific mAbs (cat # IM3497, IO TEST Beta Mark TCR-Vβ repertoire kit, Beckman Coulter) used in association with CD3-specific mAb (cat # 340949, BD Biosciences, 10 µL) and isotype-matched control mAbs (cat # 552834, BD Biosciences).

### Real time PCR to determine copy number of integrated CAR

To determine the copy number of integrated CD19RCD28 CAR in genetically modified T cells, 50–100 ng genomic DNA (cat # 80204, AllPrep DNA/RNA Mini Kit, Qiagen) was amplified using Steponeplus Real-time PCR system (Applied Biosystems) in a PCR reaction (2 min for 50°C, 10 min for 95°C, followed by 40 cycles of 15 sec 95°C and 1 min 60°C) using the following primers: forward (5′-CAGCGACGGCAGCTTCTT-3′), reverse (5′-TGCATCACGGAGCTAAA-3′) and probe (5′-AGAGCCGGTGGCAGG-3′). Primers (Cat # 4316844, Applied Biosystems) for RNAse P gene were used as an internal control. Serially-diluted genomic DNA from a genetically modified Jurkat-cell (clone # 12) containing 1 copy of CAR from _CoOp_CD19RCD28/pSBSO DNA plasmid was used to generate a standard curve [20]. All the primers, probes and TaqMan Gene Expression Master Mix were purchased from Applied Biosystem.

### PCR for SB11 transposase

DNA (20 ng) (AllPrep DNA/RNA Mini Kit, Qiagen) isolated from CAR^+^ T cells was amplified using a thermal cycler (PTC-200, DNA Engine, BioRad) using forward (5′-ATGGGAAAATCAAAAGAAATC-3′) and reverse (5′-CTAGTATTTGGTAGCATTGC-3′) primers in a PCR reaction (95°C for 5 min; 25 cycles of 95°C for 15 sec, 58°C for 40 sec, 72°C for 60 sec; followed by a final extension at 72°C for 7 min). GAPDH was used as the housekeeping gene and was amplified in the same PCR reaction using the primers, forward (5′- TCTCCAGAACATCATCCCTGCCAC-3′) and reverse (5′- TGGGCCATGAGGTCCACCACCCTG-3′). Linearized pCMV-SB11 plasmid DNA (1 ng) and genomic DNA (20 ng) from genetically modified Jurkat cells stably expressing SB11 and EGFP (expressed from DNA plasmid SB11-IRES2-EGFP) [20] were used as positive control. Mock electroporated (CAR^neg^) and OKT3-aAPC-propagated T cells were used as a negative control.

### Assay to assess for unwanted autonomous T-cell growth

To monitor aberrant T-cell growth, 2×10^5^ CAR^+^ T cells, harvested after 4 aAPC-mediated stimulation cycles (28 days after electroporation) were cultured in triplicate in a 24-well tissue culture plate for an additional 18 days. (i) Positive control: the presence of aAPC and cytokines (50 U/mL IL-2 and 30 ng/mL IL-21). (ii) Test: the absence of aAPC and cytokines. The genetically modified T cells passed the assay when total viable cells at day 18 were (i) >2×10^5^ cells for CAR^+^ T cells cultured with aAPC and cytokines and (ii) <2×10^4^ cells for CAR^+^ T cells cultured without aAPC and cytokines.

### G-band karyotyping

CAR^+^ T cells at the end of co-culture were harvested and the slides were stained using Giemsa stain using standard procedure. A total of 20 G-banded metaphases were analyzed.

## Results

### aAPC (clone #4)

**Figure 1:**
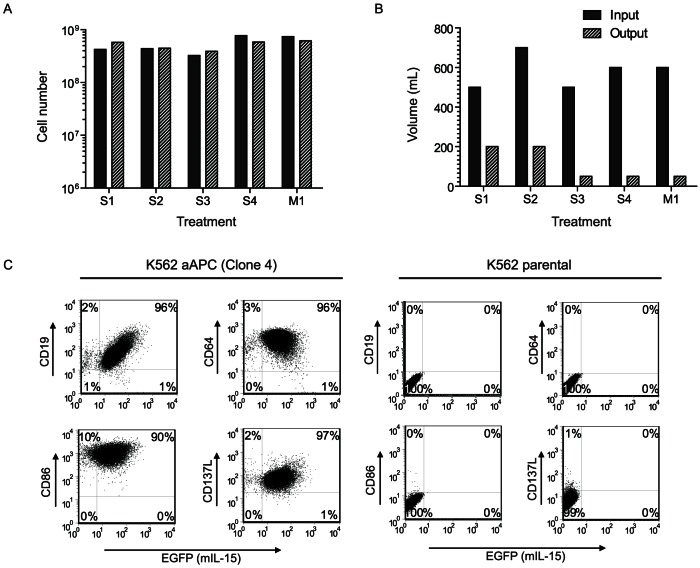
Harvest and characterization of aAPC. (A, B) Sepax volume reduction. aAPC clone #4 grown in VueLife bags were harvested using CS-490.1 kit in Sepax II. The Sepax harvest (S, n = 4) was compared to manual (M, n = 1) procedure. The mean pre/post-processing cell-counts (4.9×10^8^ vs 5×10^8^) were similar using the Sepax system. (C) Phenotype of aAPC (clone #4). Flow cytometry analysis showing expression of CD19, CD64, CD86, CD137L and mIL-15 (expressed with EGFP) (mIL-15-EGFP) on K562 aAPC and K562 parental controls.

K562 functioning as aAPC (clone #4) were employed to selectively propagate CAR^+^ T cells. The cultured aAPC were harvested from VueLife bags by Sepax II device using the volume reduction procedure which took ∼40 minutes. The mean preprocessing volume and aAPC counts were 575 mL (range 500–700 mL) and 4.9×10^8^ (range 3.2×10^8^ to 7.7×10^8^), respectively. After processing with Sepax II, mean recovery of cells was 108% (range 75 to 136%), with an output volume of 125 mL (range 50 to 200 mL) resulting in mean volume reduction of 78.3% (range 60 to 91.6%, **Figure 1A, B**). The automated cell recovery was similar to a manual volume reduction procedure (82%) which took 45 minutes of sustained operator time and resulted in 91% volume reduction. aAPC were regularly monitored by flow cytometry for >80% expression of the introduced transgenes coding for CD19, CD64, CD86, CD137L, and EGFP (as a marker for expression of mIL-15). The immunophenotyping was undertaken upon generating the MCB and WCB and upon each addition of γ-irradiated aAPC to T-cell cultures (that marked the beginning of each stimulation cycle, **Figure 1C**). MCB and WCB for Clone 4 aAPC tested negative for sterility and mycoplasma on cells and cell supernatant. In the biosafety testing, no virus was detected by adventitious virus testing, replication competent retrovirus testing, and screening for a range of human pathogenic viruses. Testing validated that the aAPC (clone #4) was derived from K562 based on finger printing (**Table S5**).

### Manufacture of CAR^+^ T cells

**Figure 2:**
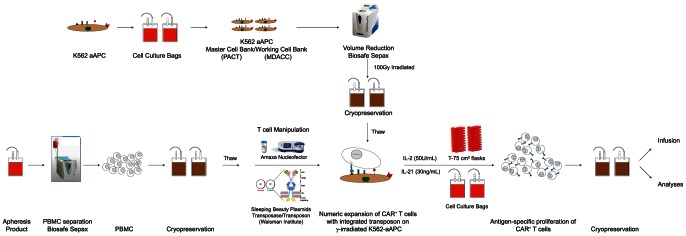
Schematic of the process of generating clinical grade CD19-specific T cells. A MCB (PACT) and WCB (MDACC) were generated for K562-derived aAPC (clone #4). For the generation of CAR^+^ T cells, aAPC were numerically expanded in bags, harvested using the Sepax II system, irradiated (100 Gy), and cryopreserved for later use. CD19-specific T cells were manufactured as follows; PBMC were isolated from normal donor apheresis products using the Sepax II system and cryopreserved. The PBMC were later thawed, electroporated with the SB DNA plasmids (CD19RCD28 CAR transposon, SB11 transposase) using the Nucleofector System, co-cultured with thawed irradiated aAPC along with cytokines (IL-2 and IL-21) for a culture period of 28 days and cryopreserved.

**Figure 3:**
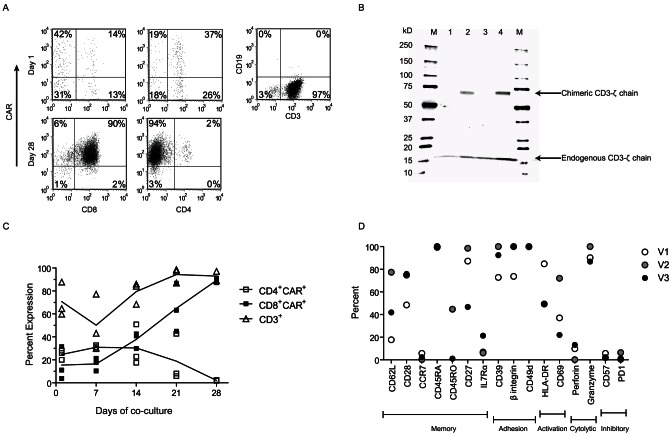
Phenotype of CAR^+^ T cell. (A) Expression of CD19RCD28 CAR on T cells day after electroporation (culture day 1) and after 28 days of co-culture on aAPC clone #4 along with lack of CD19^+^ aAPC. (B) CAR expression by western blot analysis using CD3-ζ specific antibody. Whole cell lysates were run on SDS-PAGE under reducing conditions. Molecular weight marker (M), Parental Jurkat cells (Lane 1), CD19RCD28^+^ Jurkat cells (Lane 2), CAR^neg^ control T cells (Lane 3) and CD19RCD28^+^ T cells (Lane 4). (C) Percent expression of CD3^+^, CD4^+^CAR^+^ and CD8^+^CAR^+^ T cells with in a lymphocyte gate in cultures over time. Each symbol represents a separate experiment; the solid lines are mean of the three validation experiments. (D) Immunophenotype of memory/naïve, adhesion, activation, cytolytic and exhaustion markers on CAR^+^ T cells at the end (d28) of co-culture.

**Figure 4:**
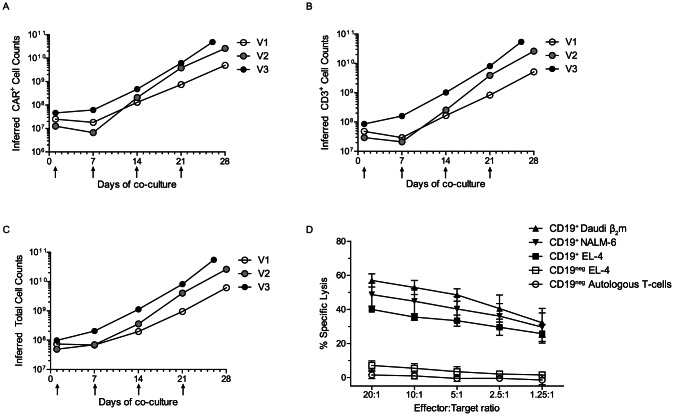
Expansion kinetics and redirected specificity of CAR^+^ T cells. Genetically modified T cells were co-cultured with aAPC clone #4 for 28 days. At the end of each stimulation cycle (7 days), cells were counted and stained for expression of CAR and CD3. Three validation runs (V1, V2, and V3) were performed and the graphs represent inferred (A) CAR^+^ T cells, (B) CD3^+^ T cells, (C) Total viable cells over time. Arrows indicate addition of aAPC to the culture. (D) Lysis of CD19^+^ targets (Daudiβ_2_m, NALM-6, CD19^+^ EL-4) as compared to background lysis of CD19^neg^ EL-4 using 4-hr chromium release assay by CAR^+^ T cells. Mean ± SD of three validation runs is represented.

Validation studies were undertaken for large scale production of CD19-specific CAR^+^ T cells to establish that PBMC can be electroporated and propagated to clinical meaningful numbers [15] (**Figure 2**) and meet pre-established release criteria (**Table 1**). Two normal donor apheresis products were processed to isolate mononuclear cells (MNC) using the Sepax cell-processing system. The apheresis products 201 mL (donor 1) and 202 mL (donor 2) were processed in two batches (∼100 mL/batch) generating a 50 mL output product. The pre-processing counts were similar to post-processing counts of the apheresis products. A total of 5.3×10^9^ and 7.1×10^9^ cells were isolated containing 40.5% and 51% CD3^+^ T cells respectively from donor 1 and donor 2. Cells were then cryopreserved in aliquots (4×10^7^/mL) in CryoMACS freeze bags (10 mL) and reference cryovials (1 mL) for later use. Three separate validation experiments were performed and are summarized in **Table 1**. For validation run 1 and 2 (V1, V2) cells from donor 1 and for validation 3 (V3) cells from donor 2 were used. For each run freshly-thawed PBMC were electroporated and *ex vivo* numerically expanded in separate culturing procedures (**Table S6**). On culture day 0, 3×10^8^ (V1) and 8×10^8^ (V2, V3) cells were thawed (viability, 88.9 to 97.6%) and rested for 2 hours prior to electroporation. 2 to 3×10^8^ cells (V1 = 2×10^8^; V2 and V3 = 3×10^8^) were electroporated at 2×10^7^ cells per cuvette with _CoOp_CD19RCD28/pSBSO transposon and pCMV-SB11 transposase DNA plasmids and the following day (culture day 1) co-cultured with aAPC clone #4 based on CAR expression. The electroporation efficiency for the three validation runs was assessed on culture day 1 as measured by expression of CAR (33.7%, 25.5% and 47.1%). CAR expression at the end of co-culture (culture day 28) was 92, 99.2 and 96.7%, and the cultures contained 95±5.3% (mean ± SD) CD3^+^ T cells with negligible amounts of contaminating CD19^+^ cells (0.7±0.15%, mean ± SD) and CD32^+^ aAPC (0.6±0.6%, mean ± SD, **Figure 3A** **,** **Table 1**). We further confirmed CAR expression by Western blot of whole-cell lysates of electroporated/propagated T cells using CD3-ζ chain-specific mAb revealing an expected 73-kDa chimeric ζ chain [10] (**Figure 3B**). Upon inspection of the kinetics of T-cell growth on aAPC, we observed an accelerated rate of T-cell propagation at the end of second week of culture (end of stimulation cycle 2), which is consistent with increased fold-expansion of total (p = 0.01) and CD3^+^ (p = 0.01) T cells as compared to the fold-expansion in the first week of stimulation. The weekly fold-expansion at the end of third and fourth week for total (p = 0.01, p<0.001), CD3^+^ (p = 0.03, p<0.001), CAR^+^ (p = 0.02, p<0.001) T cells was consistently higher than that of week-one, respectively (**Figure S4**). We observed similar weekly fold-expansion for CD3^+^ and CAR^+^ T cells past week one of stimulation. After 4 weeks of co-culture on γ-irradiated aAPC there was an average 545-fold numeric expansion of CD3^+^ T cells with a 1,111-fold expansion of CAR^+^ T cells. The *ex vivo* expansion (culture day28) resulted in an average 2.86×10^10^ CD3^+^ T cells, almost all of which were CAR^+^ (2.65×10^10^). The propagation kinetics of total (p = 0.18), CD3^+^ (p = 0.17) and CAR^+^ (p = 0.2) T cells for the three validation runs were similar (**Figure 4A, B, C**). These data support the recursive addition of aAPC for the selective outgrowth of CD19-specific T cells.

**Table 1:** Acceptance criteria for releasing electroporated and popagated T cells.

Test	Specification	Validation Run #1	Validation Run #2	Validation Run #3
**Sterility – Bacteria and Fungi**	Negative (No growth at 14 days)	No growth at 14 days	No growth at 14 days	No growth at 14 days
**Mycoplasma**	Negative by PCR	Negative	Negative	Negative
**Visual Inspection**	No evidence of contamination	No evidence of contamination	No evidence of contamination	No evidence of contamination
**Viability (Trypan Blue or 7AAD)**	≥70%	99%	99%	98%
**Gated CD3^+^ Immunophenotyping**	≥80%	89%	97%	99%
**Gated CAR^+^ Immunophenotyping**	≥10%	92%	99%	96%
**Gated CD32^+^ Immunophenotyping**	<5%	1.3%	0.04%	0.51%
**Gated CD19^+^ Immunophenotyping**	<5%	0.75%	0.49%	0.78%
**Endotoxin LAL**	Endotoxin level <5 EU/recipient Weight (For validation, per mL)	<0.400 EU/mL	<0.667 EU/mL	<0.089 EU/mL
**Screen for unwanted Autonomous Growth**	<2×10^4^ cells for the cells without cytokines or aAPC at Day 18	<2×10^4^ cells	<2×10^4^ cells	<2×10^4^ cells

### Immunophenotype of electroporated and propagated CAR^+^ T cells

Two of the three validation runs resulted in a preferential growth of CD8^+^CAR^+^ T cells (76±22%, mean ± SD) as compared to CD4^+^CAR^+^ T cells (16±24%, mean ± SD) (**Figure 3C**) which was predicted by inclusion of IL-21 [11]. Mean CD4/CD8 ratios for CAR^+^ T cells modulated during the course of the co-culture as there was a predominance of CD4^+^CAR^+^ T cells at the start of the co-culture on aAPC (culture day1, ratio = 3.3) leading to equal amounts of CD4^+^CAR^+^ and CD8^+^CAR^+^ T cells at culture day 14 (ratio = 0.9), after which the ratio declined (culture day 21, ratio = 0.4; culture day 28, ratio = 0.3). The total CD4/CD8 ratio followed a similar trend and declined over time (**Table S6**). The CAR^+^ T cells at the end of the propagation period (culture day 28) were activated (CD69 expression, mean 43.6%; HLA-DR expression, mean 61.2%), capable of cytolysis (Granzyme B expression, mean 92.3%) and expressed markers of memory/naïve T cells (CD62L expression, mean 45.6%; CD28 expression, mean 66.4%; CD27 expression, mean 77.5%, CD45RA expression, mean 99.7%). We were not able to detect cell-surface markers of exhaustion/senescence (PD-1 expression, mean 2.7%; CD57 expression, mean 3.3%) (**Figure 3D**). These data are consistent with the aAPC supporting the outgrowth of a heterogeneous population of CAR^+^ T cells.

### Redirected specificity of CAR^+^ T cells

T cells generated from all three validation runs were able to specifically lyse CD19^+^ tumor targets. An average of 57±4% (mean ± SD; range, 61.2 to 53.8%) Daudiβ_2_m and 49±7% (mean ± SD; range, 41 to 54%) NALM-6 was lysed at an effector to target ratio of 20∶1. CD19-specific killing was demonstrated by 6.2±2.6 (mean ± SD)-fold higher killing of CD19^+^ EL-4 (range, 4.2 to 9.2 fold) as compared to the CD19^neg^ parental EL-4 cells at effector/target ratio of 20∶1 with a 1.4±1 (mean ± SD)-fold background CD19-specific lysis by CAR^neg^ (mock electroporated) controls (**Figure 4D** **, Figure S5**). This implies that the CAR in the electroporated and propagated T cells redirected killing to CD19.

### Lack of unwanted autonomous proliferation by genetically modified and propagated T cells

We evaluated growth of CAR^+^ T cells in the presence/absence of K562 aAPC and cytokines (IL-2 and IL-21) to rule out aberrant T-cell growth due to potential genotoxicity caused by SB transposition. At the end of 18 days of culture we observed <2×10^4^ cells (mean 2,800, range 0.0–5.6×10^3^) in the genetically modified T cells receiving no cytokines and aAPC, while the control group receiving cytokines and aAPC numerically expanded to an average of 7.6×10^7^ cells, range 4.12×10^7^ to 12.8×10^7^ (**Table 2**). These data indicate CAR^+^ T cells cannot sustain proliferation upon withdrawal of growth factors and antigenic stimulation.

**Table 2:** Lack of autonomous cell growth by genetically modified T cells.

Experiment	Day of culture^a^	No. of T-cells seeded^b^	No. of T cells (d18) without cytokines and aAPC^c^	No. of T cells (d18) with cytokines and aAPC^d^	% fold-change of T-cells^e^
V1	28	2×10^5^	0	12.80×10^7^	0.000%
V2	28	2×10^5^	5.6×10^3^	5.88×10^7^	0.009%
V3	28	2×10^5^	2.8×10^3^	4.12×10^7^	0.007%

a Days of culture when T cells were seeded.

b Total number of T cells seeded in culture at the start of the experiment.

c Total number of T cells counted in the absence of cytokines and aAPC.

d Total (inferred) number of T cells counted in the presence of cytokines and aAPC (positive control).

e Percent fold-change = [(c/b)÷(d/b)]*100.

### Telomere length in CAR^+^ T cells

**Figure 5:**
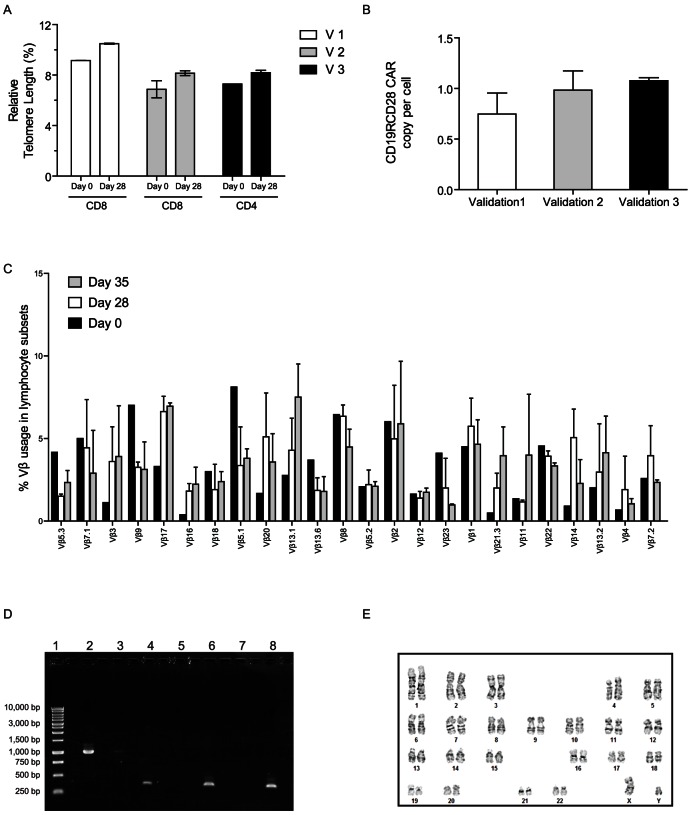
Safety profile associated with the SB system. (A) Telomere length of cells was measured using fluorescence in situ hybridization and flow cytometry (Flow-FISH) assay. Predominant T cell population at day 28 (V1 and V2, CD8^+^ T cells; V3, CD4^+^ T cells) was compared to respective miltenyi column purified subset of T cells from day 0. Mean ± SD of triplicates for each validation run is represented. (B) Genomic DNA from CAR^+^ T cells at day 28 was amplified using primers and probes specific for CD19RCD28 CAR. Relative Quantity (RQ) analyses of the CD19RCD28 target copy number was determined using normal donor PBMC as reference and endogenous RNaseP as a normalizer. Mean ± SD of triplicates for each validation run is shown. (C) TCR Vβ analysis of day 28 and day 35 CAR^+^ T cells. Data shows mean ± SD of three validation run CAR^+^ T cells as compared to day 0 unmanipulated controls. (D) A representative genomic PCR showing lack of SB11 transposase integration. Genomic DNA (20 ng) was amplified using SB11 or GAPDH primers. CAR^neg^ control T cells (lane 5) and CAR^+^ T cells (lane 7) amplified using SB11 primers; CAR^neg^ control T cells (lane 6), CAR^+^ T cells (lane 8) and Jurkat stably expressing SB11 (lane 4) amplified using GAPDH primers. Jurkat stably expressing SB11 (Jurkat/SB11-IRES2-EGFP) (lane 3) and the linearized plasmid, pKan-CMV-SB11 (lane 2) amplified using SB11 primers were used as positive controls. (E) G-banded karyotypes of CAR^+^ T cells from the three validation runs reveal no structural or numeric alteration. A representative spread from validation 2 is shown.

Telomere length is an important measure of cellular differentiation and progression to senescence. Therefore to evaluate the effect of SB transposition and *ex vivo* numeric expansion of CAR^+^ T cells on telomere length, we compared telomere lengths from CAR^+^ T cells (culture day 28) to their respective matched unmanipulated controls (prior to electroporation) using Flow-FISH assay. Due to the generation of predominately CD8^+^ CAR^+^ T cells in V1 and V2 and CD4^+^ CAR^+^ T cells in V3, we compared telomere lengths of CD8^+^ T cells for V1 & V2 and CD4^+^ T cells for V3 to CD8^+^ and CD4^+^ T cells, respectively from prior to propagation (**Figure 5A**). The average telomere length of T cells after *ex vivo* numeric expansion (culture day 28, 8.93±1.33%; mean ± SD) was similar to that of unmanipulated control T cells (day 0, 7.77±1.21%; mean ± SD). These results indicate that electroporation and propagation of CAR^+^ T cells does not result in erosion of telomere lengths.

### Copy number of CAR transgene

The copy number of integrated CD19RCD28 transgenes was determined using CD19RCD28^+^ Jurkat cell clone # 12 as reference and endogenous RNase P as a normalizer [20]. The average transgene copy per T cells generated in the validation runs was 0.96±0.09 (mean ± SD; range, 0.75 to 1.07, **Figure 5B**). These data indicate that SB transposition resulted in approximately one integrated copy of CAR per T-cell genome.

### TCR Vβ usage

The electroporation and propagation of T cells may lead to emergence of oligoclonal or clonal population of T cells that could be indicative of preferential growth and thus an indicator of a genotoxic event. Therefore, we evaluated TCR Vβ usage by flow cytometry as a means to assess repertoire diversity. All 24 TCR Vβ families tested were preserved in T cells after 28 days of co-culture on aAPC and similar to the pre-electroporation repertoire. Further, the TCR Vβ families were preserved upon prolong culture time (culture day 35, **Figure 5C**). These data suggest maintenance of a broad TCR diversity in cultured CAR^+^ T cells and do not reveal an imbalance in the use of TCR sequences.

### Lack of SB11 transposase in CAR^+^ T cells

Continued expression of SB11 transposase may lead to remobilization of the integrated CAR transgene. Therefore, we performed a genomic PCR to rule out illegitimate homologous recombination of the DNA plasmid coding for SB11 in CAR^+^ T cells. Within the limits of the assay we were unable to detect a band (∼1 kb) in PCR reactions containing DNA from CAR^+^ T cells cultured for 28 days and amplified using SB11-specific primers (**Figure 5D**). These data indicate a lack of integration of SB11 transposase in the electroporated and propagated T cells.

### Karyotype of CAR^+^ T cells

The integrity of chromosome structure was evaluated to rule out global genotoxicity associated with SB transposition. G-banding of CAR^+^ T cells (harvested 28 days after electroporation) from all the three validation runs revealed a normal (male) karyotype in all analyzed metaphase spreads (**Figure 5E** **, Figure S6**).

## Discussion

Clinical trials demonstrate anti-tumor effects in patients that have received T cells genetically modified to have desired specificity. We describe a new approach to manufacturing CD19-specific CAR^+^ T cells in compliance with cGMP. This validation study was designed to represent the process (**Figure 2**) [15] which will be used in the Phase I clinical trial [16]. This was achieved by electro-transfer of DNA plasmids derived from SB system and propagation of the genetically modified T cells on aAPC. The approach resulted in outgrowth of clinically-appealing numbers of CAR^+^ T cells that demonstrated specificity for CD19 and met release criteria (**Table 1**) established with FDA for use in clinical trials. Furthermore, in-process testing (**Table S7**) was undertaken to help reassure patient safety.

Our approach offers several advantages over other methods to generate clinical-grade CAR^+^ T cells. (i) SB transposition using DNA plasmids is a non-viral approach to gene therapy. These plasmids can be produced at reduced expense compared with clinical-grade recombinant virus used to transduce T cells. Furthermore, large amounts of DNA plasmids can be produced for human application by many academic and industrial groups which by-passes the need to rely on a few facilities expert in manufacturing virus in compliance with cGMP. The use of DNA plasmids thus facilitates the design and testing of new CARs, for example that can activate T cells through CD137 instead of (or in addition to) CD28. (ii) The pre-irradiated and cryopreserved aAPC can be used as an “off-the-shelf” reagent replacing the need to procure PBMC as allogeneic feeder cells. This saves time and expense associated with manufacturing and reduces the variability between production runs due to variation in the ability of γ-irradiated PBMC to sustain T-cell proliferation.

Our approach to manufacturing includes the implementation of processing and culturing systems to reduce work load and safeguard against a breach in sterility. To this end we co-culture T cells with γ-irradiated aAPC in bags rather than flasks. This transition typically occurs after day 14 following electroporation. In addition, the aAPC as source material are numerically expanded in bags and we have adapted the Sepax device to process the aAPC for cryopreservation. The Sepax harvest procedure has additional advantage beyond automation when using large volumes of culture media (>900 mL) as it reduces additional centrifuging steps required by the manual process.

Our manufacturing validation data set are used in support of two clinical trials to infuse patient-derived and donor-derived CD19-specific T cells after autologous and allogeneic HSCT [16]. These trials, which are currently accruing research participants, will infuse T cells after delivery of standard-of-care conditioning regimen and administration of hematopoietic stem cells. The trials are powered to assess the safety, feasibility and persistence of the infused T cells. Additional studies will comment on the potential for an anti-tumor effect, and immune response against CD19RCD28, emergence of sub-population of T cells that have sustained proliferation/survival *in vivo*, and ability of the CAR^+^ T cells to home to sites of disease. These studies are aided by our ability to detect the CAR using a proprietary monoclonal antibody that binds the scFv region [21] and the development of Q-PCR using CAR-specific primers to detect the CAR [20].

In aggregate the data reported were used to help secure institutional and federal (NIH-OBA and FDA) approvals for the two trials.

## Supporting Information

Figure S1 **Generation of CD19RCD28 CAR transposon.** The _CoOp_CD19RCD28/pEK vector containing a codon optimized chimeric antigen receptor (CAR) and SB DNA plasmid pT-MNDU3-EGFP [10], [22] were digested with *Spe*I & *Nhe*I and *Spe*I & *Nru*I to release CAR and EGFP fragments respectively. The EGFP-deleted pT-MNDU3 vector was then ligated with CAR fragment to generate _CoOp_CD19RCD28/pT-MNDU3 vector. Further the Kanamycin resistance gene (Kan^R^) and the ColE1 origin of replication obtained by *Ase*I & *Pac*I digestion of pEK vector was ligated into *Sal*I & *Zra*I digested _CoOp_CD19RCD28/pT-MNDU3 vector to create Pre- _CoOp_CD19RCD28/pSBSO. In the last step, MNDU3 promoter from Pre- _CoOp_CD19RCD28/pSBSO was released using digestion with *Nhe*I & *Nsi*I and replaced with hEF-1α promoter fragment obtained from pVitro4 vector using *Xho*I & *Nhe*I, to generate the final vector _CoOp_CD19RCD28/pSBSO.
(EPS)

Figure S2 **Generation of SB11 transposase.** SB transposase vector pCMV-SB11 was digested *with Pvu*II to release the fragment containing CMV promoter/enhancer and SB transposase encoding gene, which was ligated to fragment containing Kanamycin resistance gene (Kan^R^) and ColE1 origin of replication from pEK vector to generate pKan-CMV-SB11 vector.
(EPS)

Figure S3 **Schematic of CD19 expression plasmid, ΔCD19CoOp-F2A-Neo/pSBSO.** The DNA fragment encoding CD19RCD28 CAR from the plasmid _CoOp_CD19RCD28/pSBSO was swapped with DNA fragment encoding neomycin resistance gene (Neo^R^) [PCR cloned from pSelect-Neo (InvivoGen)] fused to codon-optimized (GENEART) truncated CD19 (ΔCD19, [23], [24]) via a F2A linker [amino acid, VKQTLNFDLLKLAGDVESNPGP; [25]–[27]] to generate ΔCD19CoOp-F2A-Neo/pSBSO. EF1α promoter, Elongation factor-1α promoter; Neo^R^, Neomycin resistance gene; bGHpAn, polyadenylation signal from bovine growth hormone; ColE1, ori; Kan^R^, Kanamycin resistance gene; IR, SB-inverted/direct repeats.
(EPS)

Figure S4 **Rate of numeric expansion of CD19-specific CAR^+^ T cells.** Genetically modified T cells were co-cultured with aAPC in a 7-day stimulation cycle, and weekly fold-expansion rate from each validation run at the end of each stimulation cycle for total, CD3^+^ and CAR^+^ T cells was calculated. Mean fold-expansion is shown (n = 3).
(EPS)

Figure S5 **Redirected specificity of CD19-specific CAR^+^ T cells.** CD19-specific lysis of CD19^+^ tumor targets (Daudiβ_2_m, NALM-6, CD19^+^ EL-4) by CAR^+^ T cells generated in three validation runs (V1, V2, V3) in a standard 4-hr chromium assay. Background autologous lysis against the CAR^neg^ control was 1.5%.
(EPS)

Figure S6 **Safety regarding chromosomal aberration.** G-banded karyotypes of CAR^+^ T cells generated from validation runs (V1 and V3) reveal no structural or numeric alteration.
(EPS)

Table S1 **Release criteria for DNA plasmids coding for SB transposon and transposase.**
(DOCX)

Table S2 **Release criteria for K562-derived aAPC (clone #4) master cell bank.**
(DOCX)

Table S3 **Release criteria for K562-derived aAPC (clone #4) working cell bank.**
(DOCX)

Table S4 **Antibodies used for flow cytometry.**
(DOCX)

Table S5 **STR fingerprinting of K562 aAPC (Clone#4).**
(DOCX)

Table S6 **Characterization of T cells before and after co-culture on γ-irradiated aAPC.**
(DOCX)

Table S7 **In-process testing for electroporated and propagated T cells.**
(DOCX)
