# Supplementary material for: Manufacture of Clinical-Grade CD19-Specific T Cells Stably Expressing Chimeric Antigen Receptor Using Sleeping Beauty System and Artificial Antigen Presenting Cells
Source: PLoS One. 2013 May 31;8(5):e64138. doi: 10.1371/journal.pone.0064138 (PMC3669363; doi:10.1371/journal.pone.0064138)
Supplement: Table S1 — Release criteria for DNA plasmids coding for SB transposon and transposase. (DOCX) [file pone.0064138.s007.docx]

**Table S1**: Release criteria for DNA plasmids coding for SB transposon and transposase

| Test | Specification |
| --- | --- |
| Appearance | Clear colorless liquid |
| Restriction Mapping & Agarose Gel | Digest with Nde I; AflIII & NheI; HindIII & AvrII; Xba & NcoI for transposon. Digest with NdeI; XhoI & PvuII; AflIII; NcoI for transposase. |
| Sequencing | Sequence conforms to original coding |
| Concentration via absorbance | 2.0±0.2mg/mL |
| A260/A280 absorbance ratio | 1.8-2.0 |
| Kinetic LAL test for Bacterial Endotoxin | <50EU/mg |
| Plasmid Form (% supercoiled via HPLC) | >90% supercoiled |
| Sterility Test | No Growth observed |
| *E.coli* host protein via ELISA | <0.3% |
| *E.coli* RNA via HPLC | <10% |
