# Supplementary material for: Manufacture of Clinical-Grade CD19-Specific T Cells Stably Expressing Chimeric Antigen Receptor Using Sleeping Beauty System and Artificial Antigen Presenting Cells
Source: PLoS One. 2013 May 31;8(5):e64138. doi: 10.1371/journal.pone.0064138 (PMC3669363; doi:10.1371/journal.pone.0064138)
Supplement: Table S2 — Release criteria for K562-derived aAPC (clone #4) master cell bank. (DOCX) [file pone.0064138.s008.docx]

**Table S2:** Release criteria for K562-derived aAPC (clone #4) master cell bank

| Test | Specification |
| --- | --- |
| Bacteriostasis & Fungistasis | Negative |
| Sterility by 21CFR610.2 | Negative |
| Endotoxin LAL | Negative |
| Agar Cultivable and Non-Agar Cultivable Mycoplasma | Negative |
| Replication-competent Lentivirus | Negative |
| Product Enhanced Reverse Transcriptase for Detection of Retrovirus | Negative |
| HIV-1/2 Proviral DNA by PCR | Negative |
| HBV DNA by PCR | Negative |
| HCV RNA by RT-PCR | Negative |
| CMV DNA by PCR | Negative |
| Parvovirus B19 | Negative |
| HTLV-I/II Proviral DNA by PCR | Negative |
| EBV DNA by PCR | Negative |
| HHV-6 DNA by PCR | Negative |
| HHV-7 DNA by PCR | Negative |
| HHV-8 DNA by PCR | Negative |
| Adeno-associated Virus | Negative |
| *In vivo* Inapparent Virus | Negative |
| *In vitro* Adventitious Virus | Negative |
| Bovine Virus by 9CFR | Negative |
| Porcine Virus by modified 9CFR PT-1 | Negative |
| Adeno-associated Virus | Negative |
| Isoenzyme analysis | Human Origin |
| Morphology by Transmission Electron Microscopy | No identifiable virus-like particles nor any other microbial agents |
| Immunophenotyping |  |
| CD19 | 90% |
| CD64 | 90% |
| CD86 | 90% |
| CD137L | 90% |
| EGFP-mIL-15 | 90% |
