# Supplementary material for: Manufacture of Clinical-Grade CD19-Specific T Cells Stably Expressing Chimeric Antigen Receptor Using Sleeping Beauty System and Artificial Antigen Presenting Cells
Source: PLoS One. 2013 May 31;8(5):e64138. doi: 10.1371/journal.pone.0064138 (PMC3669363; doi:10.1371/journal.pone.0064138)
Supplement: Table S3 — Release criteria for K562-derived aAPC (clone #4) working cell bank. (DOCX) [file pone.0064138.s009.docx]

**Table S3:** Release criteria for K562-derived aAPC (clone #4) working cell bank

| **TEST** | **LABORATORY** | **SPECIFICATION** |
| --- | --- | --- |
| **Bacteriostasis and Fungistasis** | AppTec Laboratory Services | Negative |
| **Sterility by 21CFR610.12** | AppTec Laboratory Services | Negative |
| **Agar Cultivable and Non- Agar Cultivable Mycoplasma** | BioReliance | Negative |
| ***In vitro* Adventitious Virus testing** | BioReliance | Negative |
| **Identity** **Isoenzyme analysis** | BioReliance | Human Origin |
| **GFP^+^ Phenotype** | MDACC GMP Flow Cytometry Laboratory | ≥80% |
| **CD19^+^ Phenotype** | MDACC GMP Flow Cytometry Laboratory | ≥80% |
| **CD86^+^ Phenotype** | MDACC GMP Flow Cytometry Laboratory | ≥80% |
| **CD137L^+^ Phenotype** | MDACC GMP Flow Cytometry Laboratory | ≥80% |
| **CD64^+^ Phenotype** | MDACC GMP Flow Cytometry Laboratory | ≥80% |
| **CD32^+^ Phenotype** | MDACC GMP Flow Cytometry Laboratory | ≥80% |
| **Endotoxin (LAL) EndoSafe** | MDACC GMP Quality Control Laboratory | ≤5EU/mL |
| **Viability By Trypan Blue** | MDACC GMP Quality Control Laboratory | ≥ 60% |
