# Supplementary material for: Manufacture of Clinical-Grade CD19-Specific T Cells Stably Expressing Chimeric Antigen Receptor Using Sleeping Beauty System and Artificial Antigen Presenting Cells
Source: PLoS One. 2013 May 31;8(5):e64138. doi: 10.1371/journal.pone.0064138 (PMC3669363; doi:10.1371/journal.pone.0064138)
Supplement: Table S4 — Antibodies used for flow cytometry. (DOCX) [file pone.0064138.s010.docx]

**Table S4**: Antibodies used for flow cytometry.

| **Antibody/Fluorochrome** | **Vendor** | **Catalogue No.** |
| --- | --- | --- |
| **T cells** | | |
| CD3-PE | BD Biosciences | 347347 |
| CD4-APC | BD Biosciences | 340443 |
| CD8-PerCPCy5.5 | BD Biosciences | 341051 |
| CD16-PE | BD Biosciences | 347616 |
| CD25-APC | BD Biosciences | 555434 |
| CD28-PerCPCy5.5 | BD Biosciences | 337181 |
| CD32-FITC | BD Biosciences | 555448 |
| CD39-FITC | eBioscience | 11-0399-42 |
| CD45RA-APC | BD Biosciences | 550855 |
| CD45RO-PE | BD Biosciences | 555489 |
| CD57-FITC | BD Biosciences | 555619 |
| CD56-APC | BD Biosciences | 555518 |
| CD62L-PE | BD Biosciences | 555544 |
| CD69-PE | BD Biosciences | 555531 |
| CD127 (IL-7Ra) - Alexa Fluor 647 | BD Biosciences | 558598 |
| CD150-PE | BD Biosciences | 559592 |
| CD279 (PD-1) -PE | BD Biosciences | 557946 |
| CCR7-PerCPCy5.5 | BioLegend | 335605 |
| Granzyme B-Alexa Fluor 647 | BD Biosciences | 560212 |
| HLA-DR-PerCPCy5.5 | BD Biosciences | 339205 |
| Perforin- FITC | BD Biosciences | 556577 |
| Anti-human Fcγ-PE | Invitrogen | H10104 |
| **K562 aAPC (Clone#4)** | | |
| CD19-PE | BD Biosciences | 555413 |
| CD19-APC | BD Biosciences | 555415 |
| CD64-PE | BD Biosciences | 558592 |
| CD86-PE | BD Biosciences | 555658 |
| CD137L-PE | BD Biosciences | 559446 |
| F(ab')2 fragment of Goat anti-Mouse IgG, F(ab')2 fragment specific-PE (For OKT3 loading) | Jackson Immunoresearch | 115-116-072 |
