# Supplementary material for: Manufacture of Clinical-Grade CD19-Specific T Cells Stably Expressing Chimeric Antigen Receptor Using Sleeping Beauty System and Artificial Antigen Presenting Cells
Source: PLoS One. 2013 May 31;8(5):e64138. doi: 10.1371/journal.pone.0064138 (PMC3669363; doi:10.1371/journal.pone.0064138)
Supplement: Table S5 — STR fingerprinting of K562 aAPC (Clone#4). (DOCX) [file pone.0064138.s011.docx]

**Table S5**: STR fingerprinting of K562 aAPC (Clone#4).

| **STR** | **K562 aAPC (Clone 4)** |
| --- | --- |
| AMEL | X |
| CSF1PO | 9,10 |
| D13S317 | 8 |
| D16S539 | 11,12 |
| D18S51 | 15,16 |
| D19S433 | 14,14.2 |
| D21S11 | 29,30,31 |
| D2S1338 | 17 |
| D3S1358 | 16 |
| D5S818 | 11 |
| D7S820 | 9,11 |
| D8S1179 | 12 |
| FGA | 21 |
| TH01 | 9.3 |
| TPOX | 8,9 |
| vWA | 16 |
