# Supplementary material for: Manufacture of Clinical-Grade CD19-Specific T Cells Stably Expressing Chimeric Antigen Receptor Using Sleeping Beauty System and Artificial Antigen Presenting Cells
Source: PLoS One. 2013 May 31;8(5):e64138. doi: 10.1371/journal.pone.0064138 (PMC3669363; doi:10.1371/journal.pone.0064138)
Supplement: Table S6 — Characterization of T cells before and after co-culture on γ-irradiated aAPC. (DOCX) [file pone.0064138.s012.docx]

**Table S6:** Characterization of T cells before and after co-culture on γ-irradiated aAPC

| **Expt.** | **Apheresis** | | | | | **Day 1 after electroporation** | | | | | | **Day 28 of co-culture** | | | | | | | |
| --- | --- | --- | --- | --- | --- | --- | --- | --- | --- | --- | --- | --- | --- | --- | --- | --- | --- | --- | --- |
|  | **CD3** | **CD4: CD8** | **CD3^+^ CD4^+^** | | **CD3^+^ CD8^+^** | **CD3** | **CD4: CD8** | **CAR** | **CD3^+^ CAR^+^** | **CD4^+^ CAR^+^** | **CD8^+^ CAR^+^** | **CD3** | **CD4: CD8** | **CAR** | **CD3^+^ CAR^+^** | **CD4^+^ CAR^+^** | **CD8^+^ CAR^+^** | **Viability** | **Fold exp*** |
| **V1** | 40.5 | 1.65 | | 18.5 | 11.2 | 64.7 | 2.4 | 33.7 | 41.0 | 28.0 | 11.1 | 88.9 | 0.02 | 92.0 | 97.7 | 2.46 | 87.2 | 99% | 82.6 |
| **V2** | 40.5 | 1.65 | | 18.5 | 11.2 | 59.8 | 4.1 | 25.5 | 17.1 | 25.9 | 3.8 | 97.1 | 0.01 | 99.2 | 97.4 | 1.77 | 91.2 | 99% | 536.7 |
| **V3** | 51.2 | 1.59 | 29.1 | | 18.3 | 87.7 | 2.3 | 47.1 | 45.0 | 20.1 | 31.5 | 99.2 | 0.8 | 96.0 | 96.0 | 43.4 | 51.4 | 98% | 561.2 |

* Total (inferred) viable cells
