# Supplementary material for: Manufacture of Clinical-Grade CD19-Specific T Cells Stably Expressing Chimeric Antigen Receptor Using Sleeping Beauty System and Artificial Antigen Presenting Cells
Source: PLoS One. 2013 May 31;8(5):e64138. doi: 10.1371/journal.pone.0064138 (PMC3669363; doi:10.1371/journal.pone.0064138)
Supplement: Table S7 — In-process testing for electroporated and propagated T cells. (DOCX) [file pone.0064138.s013.docx]

**Table S7:** In-process testing for electroporated and propagated T cells.

| **Expression** | |
| --- | --- |
| Cell Surface CAR Expression | Flow Cytometry |
| Total CAR Expression | Western Blot Analysis |
| **Functionality** | |
| Cytotoxicity | Chromium Release Assay |
| **Persistence** | |
| Memory/Naïve Phenotype | Flow Cytometry |
| Telomere Length | Flow-FISH |
| **Safety** | |
| CAR Copy Number | Q-PCR |
| SB11 Detection | PCR |
| TCR Vβ Repertoire | Flow Cytometry |
| Karyotyping | G-banding |
